# Differences in the risk of mood disorders in patients with asthma-COPD overlap and in patients with COPD alone: a nationwide population-based retrospective cohort study in Korea

**DOI:** 10.1186/s12931-019-1039-5

**Published:** 2019-04-30

**Authors:** Hye-Rim Kang, Sung-Hyun Hong, So-Young Ha, Tae-Bum Kim, Eui-Kyung Lee

**Affiliations:** 1 School of Pharmacy, Sungkyunkwan University, 2066, Seobu-ro, Jangan-gu, Suwon-si, Gyeonggi-do 16419 South Korea; 2 Department of Allergy and Clinical Immunology, Asan Medical Center, University of Ulsan College of Medicine, Seoul, Korea

**Keywords:** Asthma-COPD overlap, Depression, Anxiety, Mood disorder

## Abstract

**Background:**

Although feelings of anxiety and depression are common in patients with chronic obstructive pulmonary disease (COPD), little is known about the estimates of their incidence in patients with asthma-COPD overlap (ACO), which has been described and acknowledged as a distinct clinical entity. We aimed to estimate the risk of depression and anxiety among patients with ACO and compare it with the risk among those with COPD alone in the general population.

**Methods:**

We conducted a nationwide population-based retrospective cohort study using the Korean National Sample Cohort database between 1 January, 2002, and 31 December, 2013. Patients who were diagnosed with COPD (International Classification of Diseases, 10th revision [ICD-10] codes J42-J44) at least twice and prescribed COPD medications at least once between 2003 and 2011 were classified into two categories: patients who were diagnosed with asthma (ICD-10 codes J45-J46) more than twice and at least once prescribed asthma medications comprised the ACO group, and the remaining COPD patients comprised the COPD alone group. Patients who had been diagnosed with depression or anxiety within a year before the index date were excluded. We defined the outcome as time to first diagnosis with depression and anxiety. Matched Cox regression models were used to compare the risk of depression and anxiety among patients with ACO and patients with COPD alone after propensity score matching with a 1:1 ratio.

**Results:**

After propensity score estimation and matching in a 1:1 ratio, the cohort used in the analysis included 15,644 patients. The risk of depression during the entire study period was higher for patients with ACO than for patients with COPD alone (adjusted hazard ratio, 1.10; 95% confidence interval, 1.03–1.18; *P* value = 0.0039), with an elevated risk in patients aged 40–64 years (1.21; 1.10–1.34; 0.0001) and in women (1.18; 1.07–1.29; 0.0005). The risk of anxiety was higher for patients with ACO than for patients with COPD alone (1.06; 1.01–1.12; 0.0272), with a higher risk in patients aged 40–64 years (1.08; 1.00–1.17; 0.0392); however, the risk was not significant when stratified by sex.

**Conclusions:**

This population-based study revealed a higher incidence of depression and anxiety in patients with ACO than in patients with COPD alone.

## Background

Chronic obstructive pulmonary disease (COPD) is a heterogeneous disease that is associated with aging and tobacco consumption; however, other exposures have also been causally related. Comorbidities contribute to the overall severity and economic burden of COPD [1]. Among such comorbidities, anxiety and depression contribute to a substantial burden of COPD-related morbidity, notably by impairing quality of life and reducing adherence to treatment [2]. In addition, recent studies have investigated the relationship between depression and anxiety with asthma, and they have shown that asthma is associated with depression and anxiety [3–5]. To date, most respiratory studies have included either patients with asthma alone (no COPD) or patients with COPD alone (no asthma) [6]. However, patients older than 40 years may present with mixed features of both COPD and asthma, which has been called Asthma-COPD overlap (ACO) [7]. Although previous studies have reported on the clinical features and poor outcomes of ACO [8, 9], there is still a debate over the defining features and disease severity of ACO [10]. Nevertheless, one of the relevance of the ACO is to identify patients with COPD who may have underlying eosinophilic inflammation that responds better to inhaled corticosteroids [9]. ACO can be useful for clinicians in terms of identifying patients with an expected poor outcome through overlapping clinical characteristics of asthma and COPD [10, 11]. Therefore, coexistence of asthma and COPD can serve as a criterion to assume ACO in a patient with COPD.

In 2017, the Global Initiative for Asthma (GINA) and Global Initiative for Chronic Obstructive Lung Disease (GOLD) committees released an updated document on the description of asthma-COPD overlap (ACO), which is characterized by persistent airflow limitation with several features usually associated with asthma and several features usually associated with COPD [7]. Compared with patients with COPD alone, patients with ACO are often considered to have different clinical manifestations, with more respiratory symptoms (such as dyspnoea and wheezing), worse health-related quality of life, more frequent exacerbations, and more comorbidities [12–14]. A previous cohort study showed a higher risk of depression in the ACO cohort compared to non-ACO cohort (adjusted HR 1.67, 95% CI 1.48–1.88) [15]. To avoid selection bias, the authors set a propensity score matched non-ACO cohort set as a comparison cohort using sex, age, and comorbidities; however, the difference in the use of ICS and oral steroid between the two cohorts remained significant. In addition, the socioeconomic status or health-care use have not been considered in the process of selecting non-ACO cohort. Therefore, selection bias still cannot be ruled out. In contrast, ACO and COPD share several common characteristics, including persistent airflow limitation and smoking, which comprise a critical diagnostic criterion and a source of these diseases, respectively [7]. Considering that asthma is a heterogeneous disease that includes patients with wide variations in the age of onset, disease severity, pulmonary function, body mass index, presence of atopy, and Th2 eosinophil inflammation [16–18], the COPD alone cohort is likely to serve as a more appropriate comparison group to achieve clinically meaningful results.

In addition, treatment options and responses may differ depending on whether a patient has COPD alone or ACO [9, 19]; therefore, it is important to determine comorbidities associated specifically with ACO and COPD. Because mood disorder is a common comorbidity in chronic respiratory diseases, including asthma and COPD [20], it is important to identify mood disorders and provide additional treatment to reduce the disease burden. However, unlike the prevalence of other comorbidities, the incidence of depression and anxiety among patients with ACO compared to COPD alone is little known. Therefore, we conducted a population-based retrospective cohort study to estimate the risks of depression and anxiety among patients with ACO and compared them with the risks among those with COPD alone.

## Methods

### Data source

This study used the National Sample Cohort data from the National Health Insurance Service (NHIS–NSC) of Korea. The NHIS uses a systematic sampling approach to randomly select a representative population of approximately 1 million people between 2002 and 2013, which is 2.2% of the total population. The sample cohort was compared with the entire population with respect to the average total annual medical expenses, residence distribution, and the mean and standard deviation of health insurance premiums; the differences were negligible during the cohort years [21]. The data gives researchers access to demographic data – including sex, age recorded at 5–year intervals, income level, and date of death – as well as the health care data – including clinical diagnoses, medical procedures, expenditures, and drug prescriptions. Information on prescribed drugs included the generic drug name, prescription date, duration, and route of administration.

### Study population

To investigate the risk of depression and anxiety in patients with ACO and patients with COPD alone, we constructed a COPD cohort using National Sample Cohort data for the period between January 2003 and December 2011. The COPD cohort included patients older than 40 years who had been diagnosed with COPD at least twice as a principal or secondary diagnosis coded according to the International Classification of Disease, tenth revision (ICD-10 codes J42, J43, and J44) and with at least 1 prescription for ≥1 of the following COPD medications: inhaled corticosteroids (ICSs), inhaled long-acting β2-agonists (LABAs), an ICS and a LABA combined in a single inhaler (ICS/LABA), inhaled short-acting β2-agonists (SABAs), inhaled long-acting muscarinic antagonists (LAMAs), short-acting muscarinic antagonists (SAMAs), a SAMA and a SABA combined in a single inhaler (SAMA/SABA), oral leukotriene antagonists, xanthine derivatives, mast cell stabilizers, and systemic corticosteroids (CSs). Within the COPD cohort, patients were divided into ACO and COPD alone groups, based on the following asthma criteria: (1) diagnosis with asthma at least twice, as a principal or secondary diagnosis (ICD-10 codes J45 and J46); (2) at least one prescription for ≥1 of the following asthma medications: ICSs, LABAs, ICS/LABA, SABAs, oral leukotriene antagonists, xanthine derivatives, mast cell stabilizers, and systemic CSs. Patients who met both criteria for COPD and asthma were defined as ACO.

**Fig. 1:**
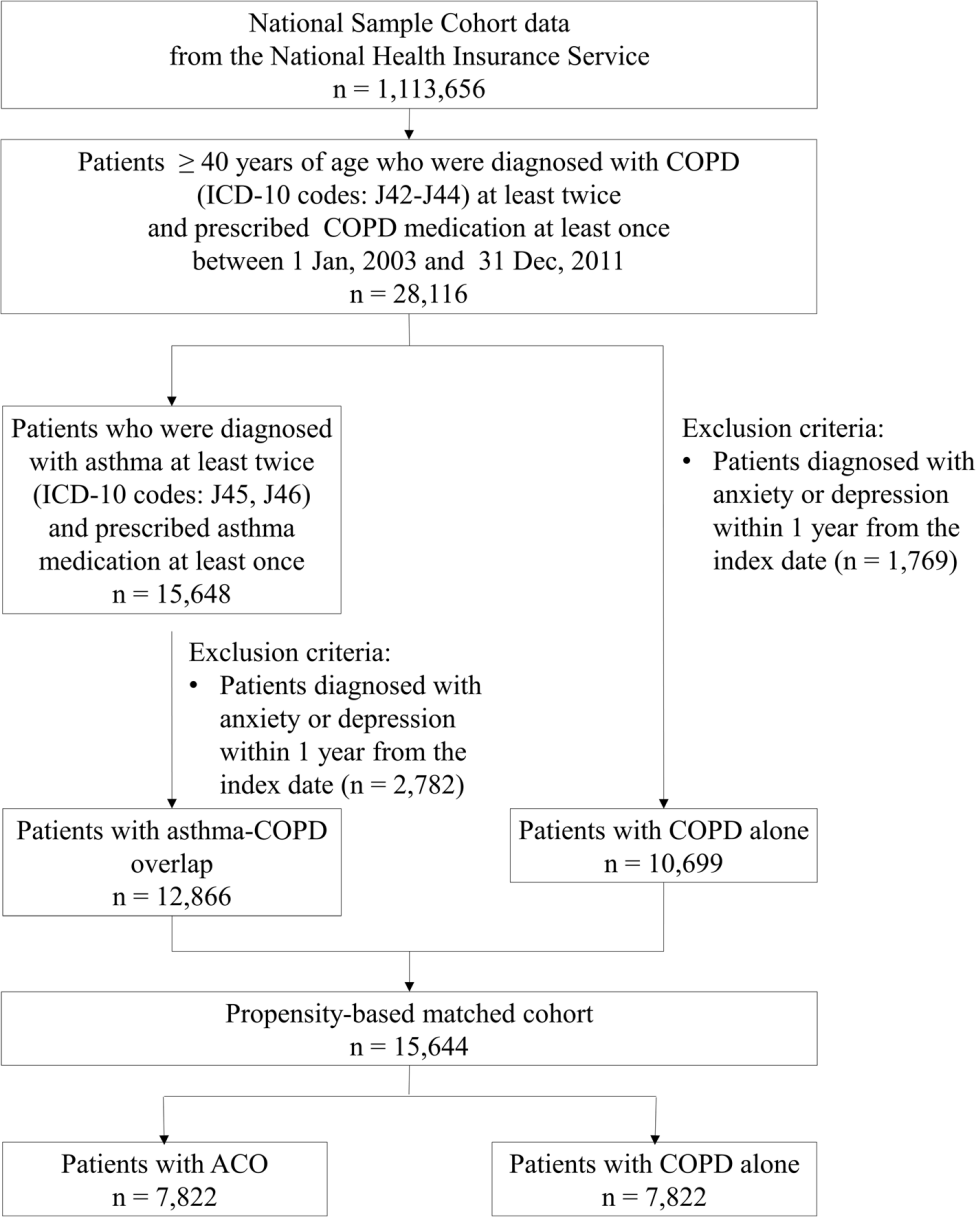
The selection of study subjects. COPD, chronic obstructive pulmonary disease; ICD-10, International Classification of Diseases; ACO, asthma-COPD overlap

Patients were excluded from the analysis of the incidence of depression and anxiety if they were diagnosed with depression or anxiety within 1 year of the index date. A study flow chart is presented in Fig. 1.

### Follow-up to depression and anxiety

**Fig. 2:**
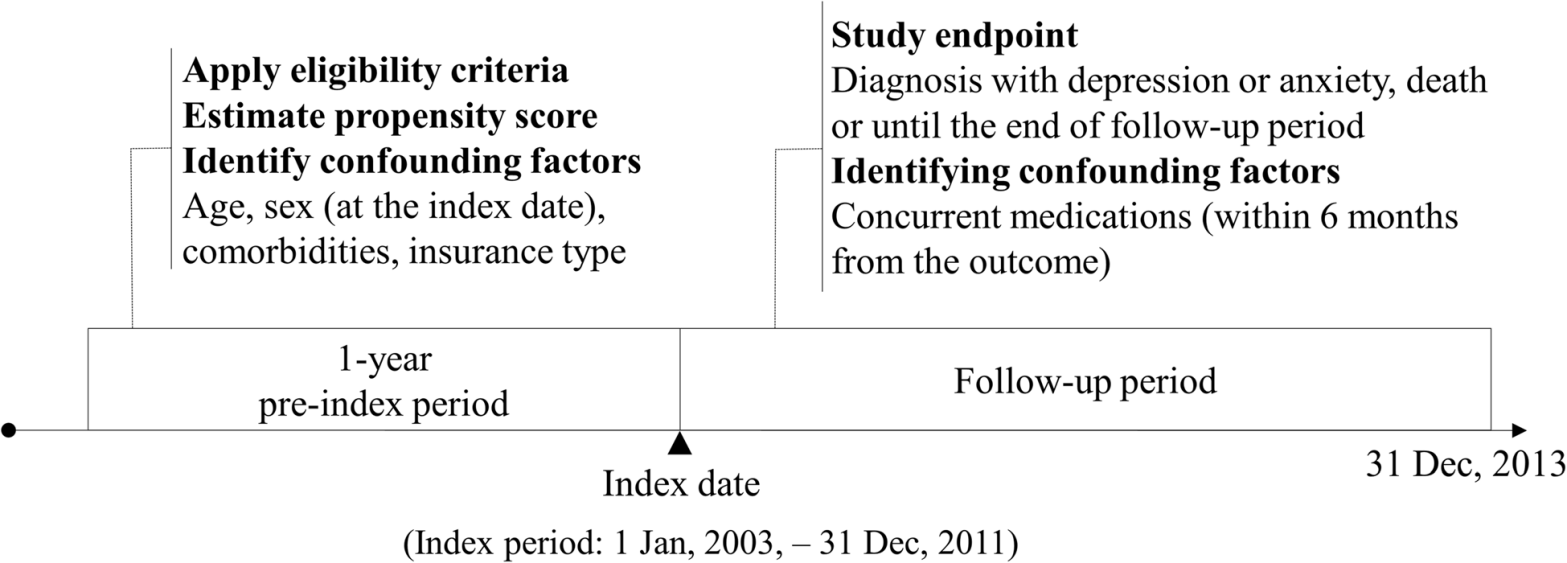
Design of the retrospective cohort study

We defined the outcome as time to first diagnosis with depression (ICD-10: F32 and F33) or anxiety (ICD-10: F40-F42) as the primary or secondary diagnosis after the index date. The index date was defined as the first date that both definitions of COPD and asthma were met. For example, if a patient with asthma met the definition of COPD later, the patient was considered eligible for inclusion in the ACO cohort from the day the patient met the definition of COPD. Patients with COPD alone were defined as those who met the definition of COPD but did not meet the definition of asthma, and the index date was defined as the first date when the patient met the definition of COPD. Follow-up was considered to have started on the index date and to have ended on the date of first diagnosis with depression or anxiety, the date the patient died, or 31 December, 2013 (Fig. 2).

### Potential confounders

Age, sex, comorbidities, and concurrent medications are all possible confounders of the association between ACO or COPD and depression or anxiety. We calculated the Charlson comorbidity index to estimate the severity of disease according to previous diagnoses within one year before the index date. We selected as confounders any comorbidities that may influence the risk of depression or anxiety, which included hypertension, diabetes, hyperlipidaemia, ischaemic heart disease, sleep disorders, alcohol-related illnesses, epilepsy, cancer, arthritis, Parkinson’s disease, dementia, obesity, cerebrovascular disease, and atherosclerosis [22]. Concurrent medications were identified based on the prescriptions within 6 months before the date of each outcome to adjust the effect of using the medications just before the occurrence of the outcomes; in analysing the incidence of depression, we used the prescription information within 6 months before the date of depression, and in the same way, we used the prescription information within 6 months before the date of anxiety in analysing the incidence of anxiety. Benzodiazepines, digitalis, and calcium antagonists – including diltiazem, nifedipine, and verapamil – were selected as confounders because they were the frequently reported medications that might increase the risk of depression or anxiety [23]. In addition, patients’ history of exacerbation and healthcare utilization were also included as potential confounders. Within the ACO and COPD cohort, patients were classified by the frequency of exacerbations they experienced within one year before the index date: frequent (≥2), infrequent (1), and not exacerbated (0). Indicators for exacerbation were identified based on ICD codes (primary diagnosis) related to COPD (or asthma in ACO) present in combination with one of the following: (1) hospitalization, (2) emergency department visit, or (3) outpatient visit with either an oral corticosteroid or antibiotic prescription within 5 days of the visit [24, 25]. To take into account for healthcare use other than for exacerbation, we included non-exacerbation related healthcare use in the preceding year of the index date as a potential confounder, which was further classified by the type of healthcare use: (1) hospitalizations, (2) emergency department visit, or (3) outpatient visit.

### Statistical analysis

We estimated the propensity scores for being defined as having ACO regardless of the outcomes by using multiple logistic regression analysis with the following variables: age category, sex, Charlson comorbidity index, history of comorbidities, exacerbations, and healthcare use in previous year, insurance type and index year. We assessed the model discrimination using the C statistic. Matching was performed using a Greedy 5 → 1 digit matching macro with the estimated propensity score [26]. We used a standardized difference to compare baseline characteristics between patients with ACO and patients with COPD alone. We defined significant difference as an absolute value greater than 0.1 [27].

We calculated the incidence rate per 1000 person-years by dividing the number of diagnoses of depression or anxiety by the total number of person-years at risk and multiplying the results by 1000. We also calculated the associated 95% confidence interval (CI). For construction of the multivariable model, we included the use of concurrent medications prescribed within 6 months before the date of the outcome as the adjusting variable. We used the Cox regression models to estimate the adjusted hazard ratios (aHRs) and their 95% CIs for depression and anxiety after adjusting for the concurrent medications in the propensity-based matched cohort.

We also conducted a subgroup analysis for the incidence of depression and anxiety according to age group, sex, comorbidities, and concurrent medications. We did subgroup analysis with interaction terms to see whether the association between ACO and incident depression or anxiety differed significantly by comorbidities and concurrent medications. All tests were 2-sided, with a significance level of 0.05. All data transformations and statistical analyses were conducted using SAS version 9.4 for Windows (SAS Institute, Cary, NC, USA).

## Results

From the NHIS-NSC database, 28,116 COPD patients aged over 40 were diagnosed with COPD more than twice between January 2003 and December 2011. Of these, we identified 15,648 patients with ACO and 12,468 patients with COPD alone. After excluding patients who were diagnosed with anxiety or depression within 1 year before the index date, 12,866 patients were included in the initial cohort of ACO and 10,699 patients were included in the initial cohort of COPD alone. After propensity score estimation and matching in a one to one ratio, the cohort used in the analysis of depression and anxiety in patients with ACO versus patients with COPD alone included 15,644 patients (Fig. 1). Table 1 shows that clinical characteristics (age, sex, Charlson comorbidity index, history of comorbidities, exacerbations and healthcare use in previous year, insurance type, and index year) were not significantly different between patients with ACO and patients with COPD alone.

**Table 1:** Comparison of clinical characteristics between patients with ACO versus patients with COPD alone

Category	No. of patients (%)		*P* value	Standardized difference^a^
	ACO (*n* = 7822)	COPD alone (*n* = 7822)		
Age group (years)				
40–64	3700(47.3)	3718(47.53)	0.7732	0.00461
≥ 65	4122(52.7)	4104(52.47)		
Sex				
Male	4170(53.31)	4176(53.39)	0.9234	0.00154
Female	3652(46.69)	3646(46.61)		
Charlson comorbidity index				
0	1436(18.36)	1537(19.65)	0.3501	0.03368
1	2645(33.81)	2603(33.28)		
2	1649(21.08)	1642(20.99)		
3	877(11.21)	855(10.93)		
≥ 4	1215(15.53)	1185(15.15)		
History of comorbidities in previous year				
Hypertension	2741(35.04)	2671(34.15)	0.2394	0.01881
Diabetes	1558(19.92)	1519(19.42)	0.4328	0.01254
Hyperlipidaemia	1319(16.86)	1313(16.79)	0.898	0.00205
Ischaemic heart disease	633(8.09)	595(7.61)	0.2586	0.01806
Sleep disorder	600(7.67)	559(7.15)	0.2107	0.02001
Alcohol-related illness	238(3.04)	225(2.88)	0.5397	0.00981
Epilepsy	67(0.86)	62(0.79)	0.6585	0.00707
Cancer	895(11.44)	874(11.17)	0.596	0.00848
Arthritis	1552(19.84)	1537(19.65)	0.7632	0.00482
Parkinson’s disease	41(0.52)	42(0.54)	0.9124	0.00176
Dementia	72(0.92)	86(1.1)	0.2629	0.0179
Obesity	3(0.04)	7(0.09)	0.2058	0.02023
Cerebrovascular disease	621(7.94)	606(7.75)	0.6555	0.00713
Atherosclerosis	168(2.15)	160(2.05)	0.6553	0.00714
History of exacerbation in previous year				
0	62(0.79)	70(0.89)	0.4844	0.01118
1	1636(20.92)	1688(21.58)	0.3095	0.01625
≥ 2	6124(78.29)	6064(77.52)	0.2476	0.01849
Healthcare utilization in previous year				
Hospitalization	1327(16.96)	1347(17.22)	0.671	0.00679
ED visit	723(9.24)	701(8.96)	0.5409	0.00978
Outpatient visit	7321(93.59)	7302(93.35)	0.5385	0.00983
Insurance type				
Health insurance	7529(96.25)	7504(95.93)	0.3022	0.0165
Medical-aid beneficiary	293(3.75)	318(4.07)		
Index year				
2003	1483(18.96)	1435(18.35)	0.3245	0.01575
2004	1107(14.15)	1032(13.19)	0.0809	0.02791
2005	910(11.63)	943(12.06)	0.4142	0.01306
2006	847(10.83)	916(11.71)	0.0811	0.0279
2007	765(9.78)	816(10.43)	0.1761	0.02163
2008	770(9.84)	809(10.34)	0.3006	0.01655
2009	768(9.82)	714(9.13)	0.1404	0.02358
2010	565(7.22)	547(6.99)	0.5754	0.00896
2011	607(7.76)	610(7.8)	0.9286	0.00143

*ACO* asthma-COPD overlap, *COPD* chronic obstructive pulmonary disease, *ED* emergency department

^a^A standardized mean difference of greater than 0.1 represents significant difference between the two cohorts

As shown in Table 2, the incidence rate of depression was 44.0 per 1000 person-years in patients with ACO and 38.2 per 1000 person-years in patients with COPD alone. The crude HR of depression among patients with ACO was 1.15 (95% CI, 1.08–1.23). After adjustment for the medications prescribed within 6 months before the date of the outcome, the adjusted HR was 1.10 (95% CI, 1.03–1.18; *P* value = 0.0039). An increased risk of depression was observed among patients aged 40–64 years with an adjusted HR of 1.21 (95% CI, 1.10–1.34; P value = 0.0001), whereas the difference in risk among patients ≥65 years was not significant between patients with ACO and patients with COPD alone. In women, the risk of depression was higher in patients with ACO (aHR, 1.18; 95% CI, 1.07–1.29; *P* value = 0.0005); however, in men, the difference in risk between ACO patients and patients with COPD alone was not significant (aHR, 1.03; 95% CI, 0.93–1.13; *P* = 0.5718).

**Table 2:** Risk of depression and anxiety in patients with ACO versus patients with COPD alone

Category	ACO (*n* = 7822)			COPD alone (*n* = 7822)			Crude HR (95% CI)	Adjusted^a^ HR (95% CI)	*P* value for adjusted HR
	Events	PY	Rate	Events	PY	Rate			
Depression									
Overall	1908	43,380	44.0	1654	43,342	38.2	1.15(1.08–1.23)	**1.10(1.03–1.18)**	**0.0039**
Age groups (years)									
40–64 (*n* = 7418)	886	22,402	39.5	727	22,817	31.9	1.24(1.13–1.37)	**1.21(1.10–1.34)**	**0.0001**
≥ 65 (*n* = 8226)	1022	20,978	48.7	927	20,525	45.2	1.08(0.99–1.18)	1.01(0.93–1.11)	0.7537
Sex									
Male (*n* = 8346)	879	22,891	38.4	792	22,448	35.3	1.09(0.99–1.20)	1.03(0.93–1.13)	0.5718
Female (*n* = 7298)	1029	20,490	50.2	862	20,894	41.3	1.22(1.11–1.33)	**1.18(1.07–1.29)**	**0.0005**
Anxiety									
Overall	3017	37,927	79.5	2780	38,155	72.9	1.09(1.04–1.15)	**1.06(1.01–1.12)**	**0.0272**
Age groups (years)									
40–64 (*n* = 7418)	1416	19,663	72.0	1318	20,155	65.4	1.10(1.02–1.19)	**1.08(1.00–1.17)**	**0.0392**
≥ 65 (*n* = 8226)	1601	18,263	87.7	1462	18,000	81.2	1.08(1.00–1.16)	1.04(0.97–1.11)	0.3314
Sex									
Male (*n* = 8346)	1394	20,536	67.9	1272	20,462	62.2	1.09(1.01–1.18)	1.06(0.98–1.14)	0.1349
Female (*n* = 7298)	1623	17,391	93.3	1508	17,693	85.2	1.09(1.02–1.17)	1.06(0.99–1.14)	0.0891

Bold results represent statistically significant

*ACO* asthma-COPD overlap, *COPD* chronic obstructive pulmonary disease, *PY* person-year; Rate, incidence rate (per 1000 person-years); HR, hazard ratio

^a^Adjusted for medications - including calcium antagonists (diltiazem, nifedipine, verapamil), corticosteroids, digitalis, and benzodiazepines - prescribed within 6 months before the date of outcome

The incidence rate of anxiety was 79.5 per 1000 person-years in patients with ACO and 72.9 per 1000 person-years in patients with COPD alone. The crude HR of anxiety in patients with ACO was 1.09 (95% CI, 1.04–1.15). After adjustment for the medication prescribed within 6 months before the date of outcome, the adjusted HR was 1.06 (95% CI, 1.01–1.12; *P* value = 0.0272). In patients aged 40–64 years, the risk of anxiety in patients with ACO was significant when compared with that in patients with COPD alone (aHR, 1.08; 95% CI, 1.00–1.17; P value = 0.0392), and ACO patients ≥65 years also did not have a significantly higher risk of anxiety (aHR 1.04; 95% CI, 0.97–1.11; P value = 0.3314). The risk of anxiety was not significant in men (aHR, 1.06; 95% CI, 0.98–1.14; P value = 0.1349), nor in women (aHR, 1.06; 95% CI, 0.99–1.14; *P* value = 0.0891).

Table 3 shows the risk of depression in subgroups according to history of comorbidities in the previous year and use of concurrent medications within 6 months before the date of outcome. We found no difference in risk associated with the comorbidities and concurrent medications, except alcohol-related illness and use of corticosteroids. The hazard ratio was higher among patients with pre-existing alcohol-related illness than those without the illness (aHR, 2.12; 95% CI, 1.44–3.12 versus aHR, 1.08; 95% CI, 1.01–1.16; P value for interaction = 0.0008). Table 4 shows the risk of anxiety according to history of comorbidities in previous year and use of concurrent medications within 6 months before the date of outcome. Any comorbidities and concurrent medications did not increase the risk of anxiety.

**Table 3:** Subgroup analyses of risk of depression in patients with ACO versus patients with COPD alone

Category	ACO (*n* = 7822)			COPD alone (*n* = 7822)			Adjusted^a^ HR (95% CI)	*P* value for interaction
	Events	PY	Rate	Events	PY	Rate		
History of comorbidities in previous year								
Hypertension								
Yes (*n* = 5412)	716	14,214	50.4	621	13,641	45.5	1.05(0.94–1.17)	0.2998
No (*n* = 10,232)	1192	29,166	40.9	1033	29,701	34.8	1.13(1.04–1.23)	
Diabetes								
Yes (*n* = 3077)	456	7961	57.3	367	7642	48.0	1.10(0.96–1.27)	0.9458
No (*n* = 12,567)	1452	35,419	41.0	1287	35,700	36.1	1.10(1.02–1.18)	
Hyperlipidemia								
Yes (*n* = 2632)	383	6425	59.6	337	6538	51.5	1.13(0.98–1.31)	0.6766
No (*n* = 13,012)	1525	36,955	41.3	1317	36,805	35.8	1.10(1.02–1.18)	
Ischaemic heart disease								
Yes (*n* = 1228)	181	3189	56.8	142	2812	50.5	1.09(0.87–1.36)	0.8601
No (*n* = 14,416)	1727	40,191	43.0	1512	40,530	37.3	1.10(1.03–1.18)	
Sleep disorder								
Yes (*n* = 1159)	228	2750	82.9	187	2546	73.4	1.10(0.91–1.34)	0.9332
No (*n* = 14,485)	1680	40,630	41.3	1467	40,796	36.0	1.10(1.03–1.18)	
Alcohol-related illness								
Yes (*n* = 463)	81	1207	67.1	39	1317	29.6	2.12(1.44–3.12)	**0.0008**
No (*n* = 15,181)	1827	42,173	43.3	1615	42,026	38.4	1.08(1.01–1.16)	
Epilepsy								
Yes (*n* = 129)	24	289	83.1	10	271	36.9	2.11(1.00–4.45)	0.0601
No (*n* = 15,515)	1884	43,092	43.7	1644	43,071	38.2	1.10(1.03–1.17)	
Cancer								
Yes (*n* = 1769)	271	4426	61.2	184	4243	43.4	1.31(1.09–1.58)	0.0562
No (*n* = 13,875)	1637	38,954	42.0	1470	39,099	37.6	1.07(1.00–1.15)	
Arthritis								
Yes (*n* = 3089)	442	8333	53.0	413	8150	50.7	0.99(0.87–1.13)	0.1019
No (*n* = 12,555)	1466	35,048	41.8	1241	35,192	35.3	1.14(1.06–1.23)	
Parkinson’s disease								
Yes (*n* = 83)	10	144	69.3	9	141	63.7	0.59(0.22–1.60)	0.5441
No (*n* = 15,561)	1898	43,236	43.9	1645	43,201	38.1	1.10(1.03–1.18)	
Dementia								
Yes (*n* = 158)	15	227	66.1	22	223	98.5	0.76(0.39–1.50)	0.1681
No (*n* = 15,486)	1893	43,153	43.9	1632	43,119	37.8	1.11(1.04–1.18)	
Obesity								
Yes (*n* = 10)	0	19	0.0	3	35	86.6	1.99(NA)	0.8967
No (*n* = 15,634)	1908	43,361	44.0	1651	43,308	38.1	1.10(1.03–1.18)	
Cerebrovascular disease								
Yes (*n* = 1227)	172	2867	60.0	157	2642	59.4	0.96(0.77–1.20)	0.1887
No (*n* = 14,417)	1736	40,514	42.8	1497	40,700	36.8	1.12(1.04–1.20)	
Atherosclerosis								
Yes (*n* = 328)	47	751	62.6	33	722	45.7	1.23(0.79–1.94)	0.7111
No (*n* = 15,316)	1861	42,630	43.7	1621	42,620	38.0	1.10(1.03–1.18)	
Use of concurrent medications within 6 months before the date of outcomes								
Calcium antagonists								
Yes (*n* = 892)	149	2150	69.3	109	1796	60.7	1.09(0.85–1.40)	0.7505
No (*n* = 14,752)	1759	41,230	42.7	1545	41,546	37.2	1.10(1.03–1.18)	
Corticosteroids								
Yes (*n* = 7658)	1192	22,314	53.4	848	18,580	45.6	1.15(1.05–1.26)	0.1887
No (*n* = 7986)	716	21,066	34.0	806	24,762	32.6	1.05(0.95–1.16)	
Digitalis								
Yes (*n* = 509)	67	1186	56.5	51	1007	50.7	1.11(0.77–1.61)	0.9571
No (*n* = 15,135)	1841	42,194	43.6	1603	42,335	37.9	1.10(1.03–1.18)	
Benzodiazepines								
Yes (*n* = 5425)	1098	13,671	80.3	943	12,750	74.0	1.08(0.99–1.18)	0.2176
No (*n* = 10,219)	810	29,709	27.3	711	30,593	23.2	1.14(1.03–1.26)	

Bold results represent statistically significant P value for interaction

*ACO* asthma-COPD overlap, *COPD* chronic obstructive pulmonary disease, *PY* person-year; Rate, incidence rate (per 1000 person-years); HR, hazard ratio

^a^Adjusted for medications - including calcium antagonists (diltiazem, nifedipine, verapamil), corticosteroids, digitalis, and benzodiazepines - prescribed within 6 months before the date of outcomes

**Table 4:** Subgroup analyses of risk of anxiety in patients with ACO versus patients with COPD alone

Category	ACO (*n* = 7822)			COPD alone (*n* = 7822)			Adjusted^a^ HR (95% CI)	*P* value for interaction
	Events	PY	Rate	Events	PY	Rate		
History of comorbidities in previous year								
Hypertension								
Yes (*n* = 5412)	1115	12,372	90.1	993	11,984	82.9	1.06(0.97–1.15)	0.977
No (*n* = 10,232)	1902	25,555	74.4	1787	26,171	68.3	1.06(0.99–1.13)	
Diabetes								
Yes (*n* = 3077)	654	6938	94.3	574	6758	84.9	1.07(0.96–1.20)	0.8967
No (*n* = 12,567)	2363	30,989	76.3	2206	31,397	70.3	1.06(1.00–1.12)	
Hyperlipidemia								
Yes (*n* = 2632)	582	5597	104.0	514	5797	88.7	1.12(1.00–1.27)	0.2248
No (*n* = 13,012)	2435	32,330	75.3	2266	32,359	70.0	1.05(0.99–1.11)	
Ischaemic heart disease								
Yes (*n* = 1228)	273	2819	96.8	202	2513	80.4	1.20(1.00–1.44)	0.155
No (*n* = 14,416)	2744	35,108	78.2	2578	35,643	72.3	1.05(0.99–1.11)	
Sleep disorder								
Yes (*n* = 1159)	314	2363	132.9	277	2095	132.2	1.02(0.86–1.20)	0.5789
No (*n* = 14,485)	2703	35,564	76.0	2503	36,060	69.4	1.06(1.01–1.12)	
Alcohol-related illness								
Yes (*n* = 463)	103	1156	89.1	82	1116	73.5	1.16(0.86–1.55)	0.6018
No (*n* = 15,181)	2914	36,771	79.2	2698	37,039	72.8	1.06(1.00–1.12)	
Epilepsy								
Yes (*n* = 129)	23	286	80.5	19	236	80.6	1.12(0.60–2.11)	0.9961
No (*n* = 15,515)	2994	37,641	79.5	2761	37,920	72.8	1.06(1.01–1.12)	
Cancer								
Yes (*n* = 1769)	351	3962	88.6	316	3710	85.2	0.98(0.84–1.15)	0.2878
No (*n* = 13,875)	2666	33,964	78.5	2464	34,445	71.5	1.07(1.01–1.13)	
Arthritis								
Yes (*n* = 3089)	718	6980	102.9	690	6866	100.5	1.00(0.90–1.10)	0.1798
No (*n* = 12,555)	2299	30,947	74.3	2090	31,290	66.8	1.08(1.02–1.15)	
Parkinson’s disease								
Yes (*n* = 83)	16	118	135.0	16	125	128.0	0.73(0.34–1.60)	0.5775
No (*n* = 15,561)	3001	37,808	79.4	2764	38,030	72.7	1.06(1.01–1.12)	
Dementia								
Yes (*n* = 158)	20	196	102.2	24	226	106.4	1.19(0.64–2.22)	0.7515
No (*n* = 15,486)	2997	37,731	79.4	2756	37,930	72.7	1.06(1.01–1.12)	
Obesity								
Yes (*n* = 10)	0	19	0.0	4	24	167.4	1.12(NA)	0.8607
No (*n* = 15,634)	3017	37,907	79.6	2776	38,131	72.8	1.06(1.01–1.12)	
Cerebrovascular disease								
Yes (*n* = 1227)	257	2435	105.5	220	2382	92.3	1.11(0.92–1.33)	0.4783
No (*n* = 14,417)	2760	35,491	77.8	2560	35,773	71.6	1.06(1.00–1.11)	
Atherosclerosis								
Yes (*n* = 328)	70	701	99.9	58	633	91.6	0.98(0.69–1.41)	0.5821
No (*n* = 15,316)	2947	37,226	79.2	2722	37,522	72.5	1.06(1.01–1.12)	
Use of concurrent medications within 6 months before the date of outcomes								
Calcium antagonists								
Yes (*n* = 859)	199	1837	108.3	152	1494	101.8	1.02(0.82–1.26)	0.3769
No (*n* = 14,785)	2818	36,090	78.1	2628	36,662	71.7	1.06(1.01–1.12)	
Corticosteroids								
Yes (*n* = 7552)	1767	18,848	93.7	1405	15,795	89.0	1.05(0.98–1.13)	0.5356
No (*n* = 8092)	1250	19,079	65.5	1375	22,360	61.5	1.08(1.00–1.17)	
Digitalis								
Yes (*n* = 511)	92	1054	87.3	76	939	80.9	1.12(0.82–1.53)	0.9439
No (*n* = 15,133)	2925	36,873	79.3	2704	37,216	72.7	1.06(1.00–1.12)	
Benzodiazepines								
Yes (*n* = 5245)	1510	10,570	142.9	1400	10,238	136.7	1.05(0.97–1.12)	0.1742
No (*n* = 10,399)	1507	27,357	55.1	1380	27,917	49.4	1.08(1.01–1.16)	

*ACO* asthma-COPD overlap, *COPD* chronic obstructive pulmonary disease, *PY* person-year; Rate, incidence rate (per 1000 person-years); HR, hazard ratio

^a^Adjusted for medications - including calcium antagonists (diltiazem, nifedipine, verapamil), corticosteroids, digitalis, and benzodiazepines - prescribed within 6 months before the date of outcome

Table 5 shows the association between the use of concurrent medications prescribed within 6 months before the date of depression or anxiety and the incidence of depression or anxiety. Among the frequently reported four types of medication that might increase the risk of depression or anxiety, calcium channel blocker, corticosteroid, and benzodiazepines were significantly associated with a higher incidence of depression or anxiety. Digitalis did not show a significant association with the incidence of depression or anxiety.

**Table 5:** Associations between the use of concurrent medications and incidence of mood disorders

Types of concurrent medications^a^	Depression		Anxiety	
	Adjusted^b^ HR (95% CI)	*P* value	Adjusted^b^ HR (95% CI)	*P* value
ACO	**1.10(1.03–1.18)**	**0.0039**	**1.06(1.01–1.12)**	**0.0272**
Calcium channel blockers	**1.30(1.15–1.48)**	**< 0.0001**	**1.14(1.02–1.27)**	**0.018**
Corticosteroids	**1.20(1.12–1.28)**	**< 0.0001**	**1.19(1.13–1.26)**	**< 0.0001**
Digitalis	1.02(0.85–1.23)	0.8139	0.88(0.75–1.02)	0.098
Benzodiazepines	**2.92(2.73–3.13)**	**< 0.0001**	**2.54(2.41–2.68)**	**< 0.0001**

Bold results represent statistically significant

*ACO* asthma-COPD overlap, *COPD* chronic obstructive pulmonary disease, *HR* hazard ratio

^a^Medications were considered as concurrent if they were prescribed within 6 months before the date of depression or anxiety

^b^Adjusted for medications - including calcium antagonists (diltiazem, nifedipine, verapamil), corticosteroids, digitalis, and benzodiazepines - prescribed within 6 months before the date of outcome

## Discussion

### Principal findings

In this population-based cohort study, we evaluated the association between ACO and the risk of depression and anxiety. Compared to patients with COPD alone, patients with ACO had a 1.10-fold increased risk of depression and 1.06-fold increased risk of anxiety. The risk of depression was higher in patients aged 40–64 years old and in women, but was not affected by presence of comorbidities within 1 year from the index date nor the use of concurrent medications within 6 months before the date of outcome, except the presence of alcohol-related illness. The risk of anxiety was higher in patients aged 40–64 years old, but was not affected by presence of comorbidities nor the use of concurrent medications. Our finding suggests that there is significant risk of depression and anxiety in patients with ACO compared with patients with COPD alone, irrespective of presence of comorbidities and use of concurrent medications.

### Comparison with other studies

Our findings are consistent with a previous cohort study in that women and patients ≥65 years of age were found to have a higher rate of depression than men and patients < 65 years of age [15]. The higher incidence of depression in our Korean study population compared to that of in the Taiwanese population can be explained by the higher prevalence of mental disorders in Korea than in Taiwan [28, 29]. In addition, our results are also similar to those from a previous study which showed the risk of depression was greater in patient with alcohol-related illness [15].

To date, our study is the first longitudinal study that has examined the incidence of anxiety disorders in patients with ACO. Our results with respect to the incidence of anxiety showed a higher rate of anxiety in women than in men, which is consistent with previously reported patterns of the prevalence of anxiety disorders in Korea [30]. In addition, a retrospective cohort study has reported that anxiety is more prevalent in patients with ACO than in those with COPD alone, with an odds ratio of 1.18 (95% CI, 1.10–1.27) [19]. Our results showed that the risk of anxiety is increased in patients with ACO compared with COPD alone. However, when we compared the risk of anxiety in ACO versus COPD patients in association with different treatments, the use of corticosteroid did not significantly increase the risk of anxiety. That is, the risk of anxiety in the patients with ACO, compared with patients with COPD alone, did not change with the use of corticosteroid.

Several studies have shown that patients with ACO have more severe respiratory symptoms, more frequent exacerbations and hospitalizations than those with COPD alone [7, 31]. In addition, COPD is treated mainly with bronchodilators, whereas ICS is recommended for the treatment of ACO patient with features of asthma [7]. Therefore, patients with ACO are not only exposed to frequent use of systemic corticosteroids due to exacerbations, but they are also more treated with regular ICS, compared to those with COPD alone. Corticosteroids exposure leading to mood disorder can be explained by the fact that chronic corticosteroid use has been associated with alterations in central and peripheral serotonin levels [32, 33]. Further studies are needed to understand the mechanism behind the higher risk of mood disorder in patients with ACO compared to those with COPD.

Association between alcohol-related illness and higher incidence of depression in patients with ACO can be explained by a research that showed acetaldehyde causing bronchoconstriction indirectly via histamine-mediated process in asthma patients [34]. Through the process of ethanol metabolism, mainly by aldehyde dehydrogenase (ALDH), ethanol is oxidized to acetaldehyde, which is further oxidized to acetate. However, many East Asian people were reported to be deficient in ALDH2, one of the ALDH isozymes [35]. When they ingest ethanol, their blood acetaldehyde and histamine levels increase significantly due to insufficient metabolic activity, and the increased histamine may result in bronchoconstriction [34, 36]. Therefore, alcohol-related illnesses in ACO patients may cause more frequent exacerbations and lead to increased risk of depression. However, why the risk of anxiety was not affected by the presence of alcohol-related illness remains unexplained, and further studies are needed.

Previous studies have shown that mood disorders cause frequent exacerbations in asthma and COPD patients [37, 38]; this can be caused by the low compliance with medication [39]. Anxiety and depression have also been associated with the activation of the hypothalamic-pituitary-adrenal axis [40], which could increase the systemic inflammatory responses and increase the risk of exacerbation. Acute exacerbation is a key indicator for assessing the degree of disease control and prognosis in patients with chronic respiratory diseases such as asthma, COPD, and ACO, because it increases mortality and lowers the quality of life [41, 42]. When an impact of the 10% increase in the relative risk of the depression is estimated in each population of 10,000 patients with ACO and with COPD alone, 1000 patients with ACO will be at risk of developing depression, compared to those with COPD alone. A previous study showed that the rate of acute exacerbation in COPD patients with mood disorders increased by 56% compared to those without mood disorders [38]. This suggests that additional 1000 ACO patients are at higher risk of developing acute exacerbations, leading to poor clinical prognosis. Therefore, our results demonstrate a need for clinicians to carefully examine for signs of mood disorders in addition to respiratory symptoms.

### Strengths and limitations

Our study has several strengths. First, to our knowledge, this is the first population-based cohort study comparing the risk of depression and anxiety between patients with ACO versus patients with COPD alone. The risk reported in previous studies was based on the comparison of patients with and without ACO, and the results showed there is a significant difference in the risk of depression between the two patient groups [15]. However, our study revealed that the increased risk is also observed in patients with ACO when they are compared with patients with COPD alone; thus, providing a basis for the importance of monitoring and paying greater attention to the signs or symptoms of depression and anxiety in patients with ACO. Second, the use of a national sample cohort database was able to yield highly representative results and overcome the possible limitations (such as insufficient statistical power) arising from a small number of patients.

Certain potential limitations should be considered when interpreting our findings. The first limitation is the definition of ACO in our study, and because this is a very contentious area already, the ACO cohort in this study was based on a subset of the COPD cohort. There is no formal definition of ACO [7]; therefore, we defined ACO based on the clinical diagnoses and corresponding prescribed medications. Although the definition of ACO has varied widely, the prevalence of ACO in our study is similar to that in previous studies [8, 12], which is estimated to be 52 to 55% of patients with COPD in database studies and 1.6 to 4.5% in the general population. The prevalence of ACO in our study was 56% (15,648/28,116) in patients with COPD and 1.4% (15,648/1,113,656) in the general population. Secondly, the measurement of outcomes was based on claims data, which does not capture patients with depression or anxiety that are not recorded in claims data (e.g. mild cases). Although there is a potential for inaccuracies in coding and for incompleteness of records, previous studies have validated the ICD-10 code-based definitions for diabetes and acute myocardial infarction (AMI), which were compared with medical records reviews and demonstrated positive predictive values of 72.3 to 87.2% for diabetes and > 70% for AMI [43, 44]. Third, residual confounding may exist due to the observational nature of this study. Several variables that could have affected the outcomes were not fully captured in the database, including smoking status, family history of mental illness, disease duration or severity, education level, and income level.

## Conclusions

The present study of a large population-based cohort study revealed that, compared with patients with COPD alone, patients with ACO have an increased risk of depression and anxiety.

## Abbreviations

ACO: Asthma-COPD overlap
COPD: Chronic obstructive pulmonary disease
CSs: corticosteroids
GINA: Global Initiative for Asthma
GOLD: Global Initiative for Chronic Obstructive Lung Disease
ICD-10: International Classification of Diseases, 10th revision
ICSs: inhaled corticosteroids
LABAs: long-acting β2-agonists
LAMAs: long-acting muscarinic antagonists
NHIS: National Health Insurance Service
NSC: National Sample Cohort data
SABAs: short-acting β2-agonists
SAMAs: short-acting muscarinic antagonists
